# Sulforaphane Repairs Oxidative Stress Damage Induced by Oxidized Fish Oil by Activating *Nrf2* in *Litopenaeus vannamei*

**DOI:** 10.1155/anu/6665220

**Published:** 2025-08-31

**Authors:** Shiping Yang, Leyuan Feng, Junliang Luo, Jichang Jian, Shuanghu Cai, Huiling Liu

**Affiliations:** Guangdong Provincial Key Laboratory of Aquatic Animal Disease Control and Healthy Culture and Key Laboratory of Control for Disease of Aquatic Animals of Guangdong Higher Education Institutes, Fisheries College, Guangdong Ocean University, Zhanjiang, China

**Keywords:** antioxidant, *Litopenaeus vannamei*, *Nrf2*, oxidized fish oil, sulforaphane

## Abstract

Nuclear factor erythroid 2-related factor 2 (Nrf2) is an essential component in regulating oxidative stress. Sulforaphane (SFN) is a natural antioxidant and gene *Nrf2* agonist that can increase the antioxidant capacity of the organism and reduce oxidative stress. However, research on the repair of oxidative stress damage by SFN in aquatic animals remains extremely scarce. In order to further explore the function and role of SFN in the repair of oxidative stress injury in aquatic animals, this study took *Litopenaeus vannamei* as the research object. We established an oxidative stress model of *L. vannamei* through 6% oxidized fish oil (OFO) feeding. Methods, such as RNA interference (RNAi), qPCR, histopathological analysis, and TUNEL assay, were used to detect the changes in the oxidative stress status of *L. vannamei*. The results showed that the expression of *Nrf2* in the hepatopancreas of *L. vannamei* in the double-stranded RNA (dsRNA)-*Nrf2* + SFN group was significantly higher than that in the dsRNA-*Nrf2* group and control group at 24 h (*p* < 0.05). The transcription levels of antioxidant and autophagy genes in the SFN group were significantly higher than those in the control group (*p* < 0.05), and the expression of related genes in the dsRNA-*Nrf2* + SFN group was also higher than that in the dsRNA-*Nrf2* group. Histopathology showed that *Nrf2* knockdown would aggravate hepatopancreatic apoptosis and vacuolation, while SFN treatment after *Nrf2* knockdown could alleviate hepatopancreatic injury and apoptosis caused by OFO. The results indicated that SFN could repair the oxidative stress injury of *L. vannamei* induced by OFO by activating *Nrf2*. This study investigated the role of SFN in alleviating and repairing the oxidative stress damage in *L. vannamei* caused by OFO, aiming to provide a theoretical basis for the research on the antioxidant effect of SFN and the regulation of the antioxidant capacity of shrimp.

## 1. Introduction

*Litopenaeus vannamei* ranks as the world's most widely farmed crustacean species and the globally preeminent penaeid in commercial value, accounting for over 80% of total global farmed shrimp production [1]. *L. vannamei* features rapid growth, relatively strong disease resistance, high aquaculture yield, and high economic value, making it one of the excellent prawn-farming varieties. However, in the process of farming, *L. vannamei* is extremely susceptible to environmental factors, such as ammonia nitrogen, nitrite, and high temperature, which leads to oxidative damage and the decline of body immunity, resulting in the outbreak of various diseases and great economic losses to the shrimp farming industry. Fish oil is a nutrient that can provide a large amount of high-quality fat and protein. It is rich in unsaturated fatty acids, which play a vital role in the growth and metabolism of aquatic animals [2]. However, these unsaturated fatty acids contain unsaturated bond structures and are extremely prone to oxidation. Fish oil is prone to being oxidized into oxidized fish oil (OFO), and it is also capable of inducing oxidative stress. Fish fed OFO showed an increasing number of free radicals, such as reactive oxygen species (ROS) [3], which can result in oxidative stress damage. Antioxidants can be appropriately added to the feed for prevention. This situation can be prevented by appropriately adding antioxidants to the feed.

Nuclear factor erythroid 2-related factor 2 (*Nrf2*) is a member of the CNC transcription factor family and contains seven homologous domains [4]. *Nrf2* performs important functions in various aspects of the body, including regulating redox balance, energy metabolism, amino acid metabolism, autophagy, apoptosis, mitochondrial function, and so on [5, 6]. *Nrf2* regulates the transcription level of over 100 genes involved in the control of apoptosis, autophagy, inflammation, antioxidant capability, and other activities, such as *NQO1*, *GSH*, *HO-1*, *CAT*, and *SOD* [6, 7]. Especially, *Nrf2* has a crucial function in cellular antioxidant protection. When the body experiences oxidative stress, the activated Nrf2 pathway can help the body remove excess ROS and reduce oxidative damage [8]. Kelch-like ECH-associated protein 1 (Keap1) is a key protein that regulates *Nrf2* activity. In cells, Keap1 can form the E3 ubiquitin ligase complex with Cullin3 (Cullin-RING E3 ubiquitin ligase) and Rbx1 (ring-box 1, E3 ubiquitin protein ligase) to regulate *Nrf2* [9]. When the body is in a normal physiological state, the ubiquitin ligase formed by Keap1 promotes ubiquitination of *Nrf2* and then degradation [9, 10]. Under oxidative stress, the ubiquitination of *Nrf2* mediated by Keap1 was prevented, the Nrf2 was translocated into the nucleus. Then *Nrf2* will bind to antioxidant response element (ARE) for the induction the gene expression of cytoprotective genes involved in antioxidant and detoxification.

Using the natural compounds as a therapeutic agent to improve oxidative stress damage is a highly promising strategy [11]. Sulforaphane (SFN) plays an important role in keeping cell homeostasis by regulating intracellular redox balance [12, 13]. Studies have shown that SFN, as a natural antioxidant, can significantly reduce oxidative damage in *L. vannamei* and enhance the immune response [14]. The oxidative stress model of *L. vannamei* induced by OFO was established to study the repair effect of SFN on oxidative damage. It is revealed for the first time that SFN can repair the oxidative stress injury induced by OFO by activating *Nrf2* of *L. vannamei*, which enriches the theoretical system of crustaceans' oxidative stress defense and provides a new idea for developing green feed additives and improving the stress resistance of shrimp.

## 2. Materials and Methods

### 2.1. Experimental Material

Shrimps were purchased from local farms (Zhanjiang, Guangdong, China). The commercial feed and fresh fish oil were provided by Guangdong Yuehai Feeds Group Co., Ltd.; SFN was purchased from Aladdin Company (Shanghai, China); and adhesives were purchased from Taizhou Hongfeng Biotechnology Co., Ltd. (Hong Jie, Taizhou, Jiangsu). Preparation of OFO based on previous research [15].

### 2.2. Preparation of Double-Stranded RNA (dsRNA)

As reported in the previous study [16], dsRNA of EGFP and *Nrf2* was synthesized using the T7 RNAi kit (Takara, China).

### 2.3. Experiment on Feed Preparation and Shrimp Culture

Based on the commercial feed with a crude protein content of 43%, a crude fat content of 5% and a crude ash content of 16%, two kinds of experimental feed for prawns were prepared: (1) OFO feed, adding 6% OFO to commercial feed, to establish the oxidative stress model of *L. vannamei*; (2) SFN feed: both OFO and 50 mg·kg^−1^ SFN were added, which was used for subsequent experiments [15]. When preparing SFN feed, firstly, 50 mg·kg^−1^ SFN and 6% OFO were added to the binder prepared in advance and mixed thoroughly [17, 18]. Next, the mix was added to the feed and mixed thoroughly again. In order to prevent the feed from deteriorating, it is stored in an environment of −20°C.

Healthy *L. vannamei* (0.75 ± 0.05 g) were selected and acclimated in the breeding bucket for 1 week to adapt to the new environment. Then divide the shrimps into 4 groups, with 3 replicates in each group and 40 shrimps in each bucket. Feed OFO regularly at 8:00, 12:00, 16:00, and 20:00 every day, and the feeding amount is calculated as 10% of the total weight of shrimp, and it is continuously fed for 35 days to induce oxidative stress. After stress induction, different groups of prawns were treated differently. Shrimps (2.07 ± 0.05 g) in the dsRNA-*Nrf2* group were injected with dsRNA-*Nrf2* (0.50 μg/g shrimp; the same below), and were fed with commercial feed. Shrimps in the dsRNA-*Nrf2* + SFN group were injected with dsRNA-*Nrf2* and supplied with SFN feed. Shrimps in the control group received dsRNA-EGFP injections and were fed the commercial feed. Shrimps in the SFN group received dsRNA-EGFP injections and were fed the SFN feed. During the experiment, the water was cleaned regularly to keep it clean. Culture water exchange, water temperature, salinity, and pH values were consistent with previous studies [14].

### 2.4. Sample Collection

The euthanasia of prawns adopts the ice-water bath method: the individual is placed in artificial seawater with aeration at 4°C (salinity 30%) until the nerve reflex is completely lost (tail limb twitching disappears), and then it is dissected and sampled quickly (refer to ISO 20178:2018, the Ethical Guide to Aquatic Animal Experiments).

At 24, 48, 72, and 96 h following the injection, the hepatopancreas of nine shrimps in each group was sampled to extract total RNA. At the same time, the hepatopancreas of shrimps were randomly sampled from each group at 96 h and fixed with Carnoy's fluid for the histopathological experiment and TUNEL detection of apoptosis.

### 2.5. Gene Expression Analysis

Total RNA was extracted from the sample tissue by RNAiso Plus reagent (TaKaRa, China), and cDNA was synthesized by PrimeScript RT kit (TaKaRa, China) containing gDNA scavenger. *EF-1α* was used as an internal reference to detect the expression of antioxidant, apoptosis, and autophagy-related genes in *L. vannamei*. The primer sequences are shown in Table 1, and the relative expression level of genes was analyzed by the 2^−ΔΔCt^ method. Using the system is the same as the previous article [19].

### 2.6. Histological and Pathological Analysis

The fixed samples were dehydrated with gradient alcohol, transparent in xylene, embedded, and cut into 8 µm slices. Following rehydration and dewaxing, sections were stained with a hematoxylin–eosin staining kit (Beyotime, China). The Leica microscope (Leica, Wetzlar, Germany) was used to view and take pictures of the stained sections.

### 2.7. TUNEL Apoptosis Detection

With the help of the TUNEL detection kit (Green FITC, Elabscience, China), hepatopancreas cell apoptosis was identified.

### 2.8. Statistical Analysis

The data were represented by the average value. The data were analyzed and tested by one-way ANOVA using IBM SPSS software. *p* < 0.05 indicates a statistically significant difference. Significant variations were shown by distinct lowercase letters.

## 3. Results

### 3.1. Expression Profile of *Nrf2* Knockdown

**Figure 1:**
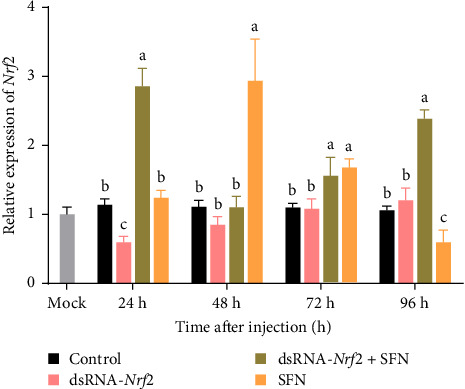
Expression of *Nrf2* in the hepatopancreas of four groups. The mock value is the expression level of the control group at 0 h. Different letters indicated significant differences at the same time (*p* < 0.05). All values are average standard deviation, *n* = 3.

As shown in Figure 1, in comparison with the control group and the dsRNA-*Nrf2* group, the transcription level of *Nrf2* in the dsRNA-*Nrf2* + SFN group was markedly upregulated at 24 h (*p* < 0.05). Specifically, it was 2.8-fold that of the control group and 4.5-fold that of the dsRNA-*Nrf2* group. The transcription level of *Nrf2* was not notable disparity between the SFN group and the control group. At 48 h, the transcription level of *Nrf2* in the SFN group was approximately 3 times that of each of the other three groups. There was no notable disparity in the expression level of *Nrf2* among the control group, the dsRNA-*Nrf2* group, and the dsRNA-*Nrf2* + SFN group at 48 h.

### 3.2. Expression of Antioxidant-Related Genes in Hepatopancreas of *L. vannamei*

**Figure 2:**
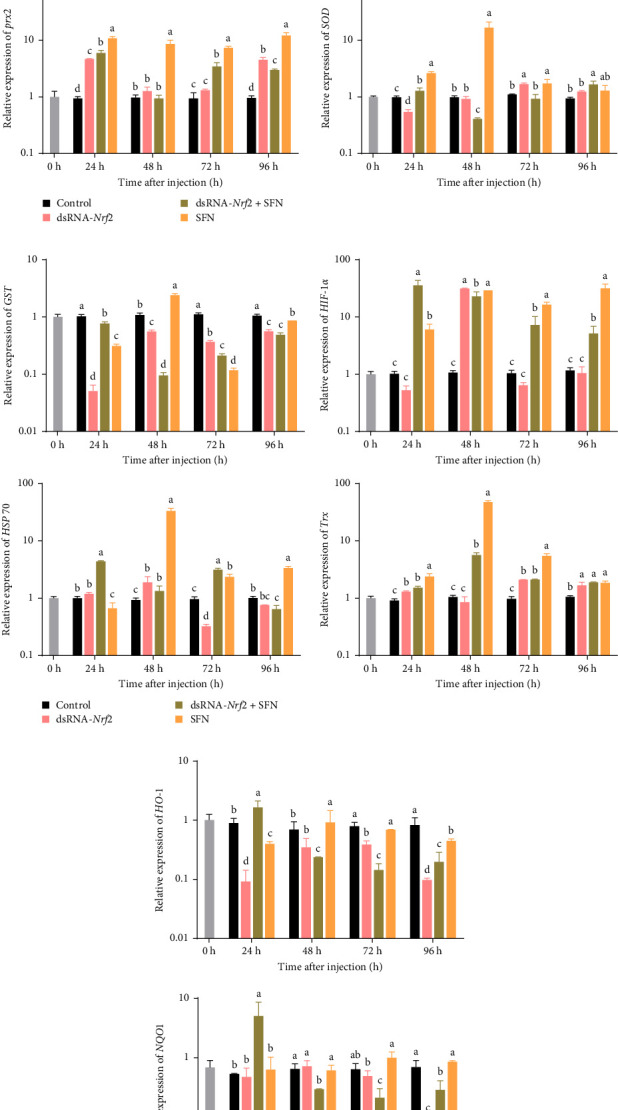
(A) The expression of direct antioxidant execution-related genes (*CAT*, *SOD*, *GPX*, and *prx2*) in hepatopancreas. (B) The expression of antioxidant pathway regulation-related genes (*HO-1* and *NQO1*) in hepatopancreas. (C) The expression of stress response and antioxidant synergistic assistance-related genes (*TRX*, *GST*, *HIF-1α*, and *HSP70*) in hepatopancreas. The different letters indicate significant differences at the same time point (*p* < 0.05).

As shown in Figure 2A–C, in contrast to the control group, the transcription level of *GST*, *SOD*, *CAT*, and *GPX* in the dsRNA-*Nrf2* group was lower (*p* < 0.05). Specifically, the expression levels of *GST*, *SOD*, and *GPX* in the control group were 3.3, 2.2, and 2.1 times those of the dsRNA-*Nrf2* group, respectively. At 24 h, in contrast to the dsRNA-*Nrf2* group, the transcription level of *HIF-1α*, *SOD*, *GPX*, *GST*, *prx2*, *NQO1*, *CAT*, and *HSP70* in the dsRNA-*Nrf2* + SFN group was significantly increased. (*p* < 0.05). Specifically, the transcription levels of *CAT*, *SOD*, *GST*, *NQO1*, *HO-1*, and *GPX* in the dsRNA-*Nrf2* + SFN group reached 4.8, 2.4, 15, 10, 2, and 5.7 times of those of the dsRNA-*Nrf2* group, respectively. The transcription level of *SOD*, *CAT*, *GPX*, *GST*, *Trx*, *HSP 70*, and *prx2* in the SFN group was the highest at 48 h,

In the dsRNA-*Nrf2* + SFN group, the expression peak of antioxidant-related genes appeared at 24 and 48 h. In the SFN group, the transcription level peak of antioxidant-related genes appeared from 48 to 96 h.

### 3.3. Expression of Autophagy and Apoptosis-Related Genes in Hepatopancreas of *L. vannamei*

**Figure 3:**
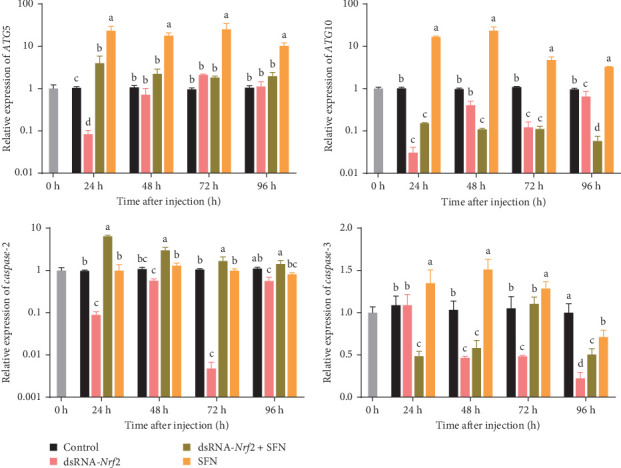
The expression of apoptosis- and autophagy-related genes in four groups of hepatopancreas. The different letters indicate significant differences at the same time point (*p* < 0.05).

As shown in Figure 3, the expression levels of *ATG5* and *ATG10* in the dsRNA-*Nrf2* group were significantly lower than the control group at 24 h (*p* < 0.05), and the expression levels of *ATG5* and *ATG10* were about 0.08 and 0.03 times those of the control group, respectively. From 24 to 96 h, the expression levels of *ATG5* and *ATG10* in the dsRNA-*Nrf2* + SFN group were stable, and the expression levels of *ATG5* and *ATG10* in the SFN group were the highest among the four groups (*p* < 0.05). From 24 to 96 h, the expression levels of *caspase-2* in the dsRNA-*Nrf2* group were unstable, while the expression of *caspase-3* gradually decreased, and the expression level of *caspase-3* was the lowest in the four groups at 96h (*p* < 0.05), which was 0.22 times the corresponding expression level of the control group. In contrast to the dsRNA-*Nrf2* group, the transcription level of *caspase-2* in the dsRNA-*Nrf2* + SFN group and the SFN group was stable and showed no significant fluctuations.

### 3.4. Pathological Analysis of Hepatopancreas

**Figure 4:**
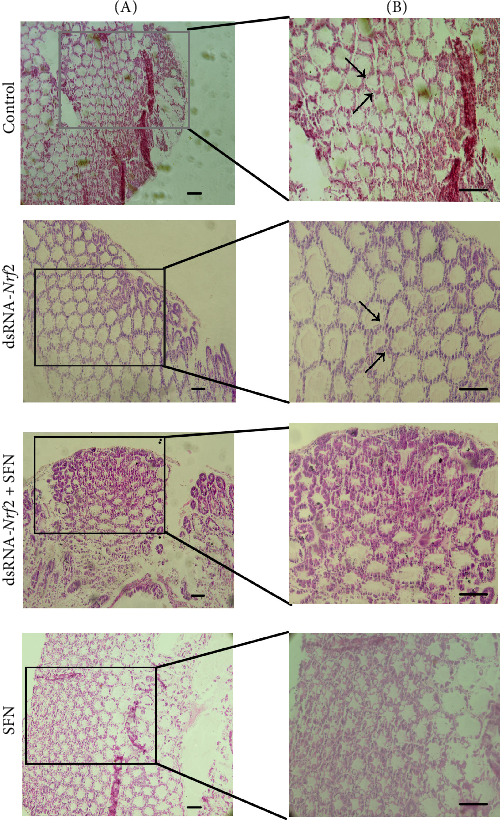
Pathological picture of hepatopancreas. Pathological pictures stained with hematoxylin and eosin at magnification of 100 times (A) and 200 times (B). Where the arrow is marked, it shows that the hepatic tubule is cavitated and the thickness of the tubular wall is reduced (scale bar: 50 μm).

As shown in Figure 4A,B, pathological analysis showed that abnormal hepatopancreatic structure appeared in the control group, including vacuolation of hepatic tubules and significant expansion of a few hepatic tubules. In the dsRNA-*Nrf2* group, not only lots of vacuoles appeared in the lumen of hepatic tubules but also the wall of hepatic tubules contracted in size. In the dsRNA-*Nrf2* + SFN group, the lumen of hepatic tubules recovered, and it showed a star shape. Moreover, in the SFN group, the degree of vacuolation decreased, and the wall thinning of hepatic tubules was alleviated. The hepatic tubules presented a well-defined star shape, and the detailed structure of the lumen was clearly visible. The results demonstrated that OFO was capable of inducing oxidative damage to the hepatopancreas. Conversely, SFN exhibited the potential to repair such oxidative damage. However, the knockdown of *Nrf2* weakened the reparative effect of SFN.

### 3.5. TUNEL Apoptosis Detection Results

**Figure 5:**
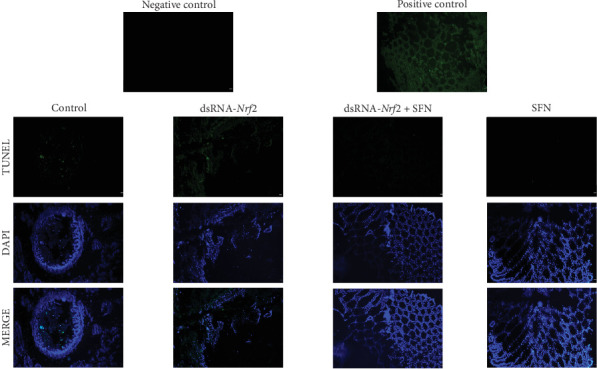
Apoptosis of hepatopancreatic cells in four groups of *L. vannamei*. The positive control is an important component in the kit used to verify the effectiveness of the detection system. The negative control, on the other hand, is used to exclude nonspecific staining and other factors that may interfere with the experimental results. Apoptotic signals are indicated by green fluorescence, with stronger green signals correlating to more severe apoptosis. The nuclei are marked with blue. All sections were examined at a magnification of 100x (scale bar: 50 μm).

As shown in Figure 5, obvious green fluorescence signals were observed in the control group. Moreover, more green fluorescent signals were observed in the dsRNA-*Nrf2* group, which indicated that apoptosis occurred in the hepatopancreas of both the control and the dsRNA-*Nrf2* groups, but the apoptosis in the dsRNA-*Nrf2* group was more serious. However, the signal of apoptosis in the dsRNA-*Nrf2* + SFN group and the SFN group was obviously weakened. The results indicated that SFN could effectively reduce the apoptosis of hepatopancreas cells.

## 4. Discussion

Our previous studies showed that *Nrf2* can regulate the antioxidant capacity, autophagy mechanism, and apoptosis of *L. vannamei* [20]. To further study the role of *Nrf2* in oxidative stress, we used OFO to induce oxidative stress in *L. vannamei* and then explored the reparative effect of SFN on oxidative stress-induced injury. Studies showed that SFN could effectively reduce oxidative damage inflicted by OFO on *L. vannamei*. This process was characterized by the significant upregulation of *Nrf2* expression, which further influenced the differential expression of antioxidant-, autophagy-, and apoptosis-related genes. The preliminary results indicated that SFN mitigated the oxidative damage of *L. vannamei* induced by OFO by activating *Nrf2*.

### 4.1. Activation of *Nrf2* by SFN and Its Antioxidant Stress Effect

When an organism undergoes oxidative stress, its antioxidant defense system will be activated. The expression level of *Nrf2* increases significantly when the organism is subjected to oxidative stress. *Nrf2* binds to the ARE, inducing the expression of antioxidant genes in the organism. Thereby, it eliminates free radicals such as ROS in the body and alleviates the oxidative stress damage caused by oxidative stress [21, 22]. SFN can not only activate *Nrf2* to reduce neuronal apoptosis and brain tissue damage, but also upregulate the expression of antioxidant-related genes through the *Nrf2* signaling pathway [23], thereby protecting the blood-brain barrier and maintaining neural function [24]. The transcription level of *Nrf2* decreased significantly 24 h after knockout, in contrast to the control group in this study. (*p* < 0.05), and no significant changes in the expression level of *Nrf2* were observed at subsequent time points. This phenomenon may be attributed to the fact that the shrimp continuously suffered from the oxidative stress induced by the intake of OFO, which maintained *Nrf2* in a highly expressed state and weakened the efficiency of RNA interference (RNAi). Compared with the dsRNA-*Nrf2* group, feeding the diet containing SFN significantly raised the transcription level of *Nrf2*. Meanwhile, the transcription level of antioxidant-related genes and stress-related genes was upregulated. Previous studies have confirmed that natural active components, such as Brassica plant extracts and dill essential oil, can significantly enhance the antioxidant capacity of aquatic animals [11–25]. Similarly, the results of this study indicated that the antioxidant capacity of the organism was further enhanced, and the damage induced by oxidative stress in *L. vannamei* was effectively alleviated and repaired after feeding the diet containing SFN.

### 4.2. The Core Regulation of Oxidative Stress and Its Epigenetic Mechanism

Although this study focuses on the Nrf2 pathway's response to oxidative stress induced by OFO, it is essential to emphasize that oxidative stress itself plays a central regulatory role in cellular regulatory networks. Recent evidence indicated that oxidative stress dynamically modulates ARE-driven gene expression through histone modifications (e.g., H3K27ac), a finding that closely aligns with the synchronous elevation of Nrf2 activity peaks and antioxidant enzyme expression observed in our study [26]. The SFN-mediated activation of Nrf2 and subsequent induction of antioxidant genes in this research essentially reflect the cell's adaptive response to oxidative stress through regulatory networks [27].

### 4.3. *Nrf2* Repairs Oxidative Stress Damage by Participating in Autophagy

Autophagy is a process in which abnormal proteins and organelles in the cytoplasm are degraded and recycled via lysosomes under stress conditions [28]. Low levels of oxidative damage can trigger autophagy, through which cells clear and renew damaged cellular components to maintain cellular and tissue homeostasis, ultimately mitigating oxidative stress-induced cellular damage [29, 30]. In this study, knockdown of *Nrf2* in *L. vannamei* significantly downregulated the expression of *ATG5* and *ATG10* at 24 h, indicating that *Nrf2* knockdown compromised the cells' ability to maintain homeostasis via autophagy under oxidative stress. SFN not only increased *Nrf2* expression in *L. vannamei* but also induced upregulation of autophagy genes *ATG5* and *ATG10*. The results indicated that SFN enhanced cellular autophagy via *Nrf2* activation to clear oxidatively damaged components, repair oxidative stress injuries, and maintain cellular homeostasis and health.

### 4.4. SFN Participates in the Regulation of Apoptosis Induced by Oxidative Stress and Its Protective Effect on Hepatopancreas

A common mode of cell death in oxidative stress is apoptosis, a programed cell death process that serves as a critical mechanism for cells to maintain homeostasis [31]. During oxidative stress, if cellular damage remains mild, cells preferentially utilize autophagy to maintain internal homeostasis. However, when the intensity of oxidative stress exceeds the cell's capacity, the autophagic mechanism becomes ineffective, and cells initiate apoptosis to eliminate irreparably damaged cells that can no longer maintain normal physiological functions, thereby preserving overall organismal health [32, 33]. *Caspase 2* acts as an initiator of programed cell death [34], while *caspase 3* serves as an apoptosis executioner [35]. In this study, *L. vannamei* with knocked-down *Nrf2* was fed a diet containing SFN. It was found that the transcription level of *caspase 2* was significantly reduced, and the difference was notable compared with the control group at 96 h. TUNEL apoptosis assay revealed that *Nrf2* knockdown increased the apoptosis of hepatopancreatic cells in *L. vannamei*, while SFN treatment significantly decreased such apoptosis. Knockdown of *Nrf2* will induce an increase in ROS and affect cell apoptosis [36, 37]. Histological analysis of the hepatopancreas demonstrated that *Nrf2* knockdown led to severe vacuolization of the hepatopancreatic lumen and thinning of the hepatic tubule walls in *L. vannamei*. Mice with *Nrf2* deficiency exacerbated the radiation-induced histopathological changes in the lungs of mice [38]. After *Nrf2* knockdown, SFN treatment significantly ameliorated the vacuolization of the tubule lumen and promoted the restoration of the hepatopancreatic tubules to their normal star-shaped morphology. Moreover, treatment with SFN alone also significantly alleviated the vacuolization of the lumen of the hepatopancreatic tubules induced by OFO, presenting a clear and complete star-shaped structure. Studies in mice have found that SFN can improve phosgene-induced lung injury through the *Nrf2* signaling pathway [39]. The results indicated that knocking down *Nrf2* exacerbated oxidative stress damage in the hepatopancreas of *L. vannamei* and led to an increase in hepatopancreatic cell apoptosis. In contrast, SFN treatment effectively mitigated oxidative stress damage in the hepatopancreas and reduced hepatopancreatic cell apoptosis. SFN may repair the oxidative stress induced by OFO in *L. vannamei* by enhancing the organism's antioxidant capacity, reducing oxidative damage, and inhibiting the initiation of apoptosis.

## 5. Conclusion

In summary, *Nrf2* knockdown exacerbated vacuolization of hepatopancreatic tubule lumens and increased cell apoptosis in *L. vannamei* fed with OFO. Conversely, SFN could activate *Nrf2* expression in *L. vannamei*, thereby regulating antioxidant-, autophagy-, and apoptosis-related gene expression and reducing apoptotic signaling in the hepatopancreas. *Nrf2* was involved in the process by which SFN repaired OFO-induced oxidative damage in *L. vannamei*.

## Figures and Tables

**Table 1:** Genes and primer sequences used in gene expression analysis.

Primer name	Sequence (5′–3′)	GenBank accession number
ds*Nrf2*-T7F	TAATACGACTCACTATAGGGAGCAACCACACATACCACAT	XM_027367068.1
dsNrf2-T7R	TAATACGACTCACTATAGGGAGGACCAACAAGATACTCCC	
ds*EGFP*-T7F	TAATACGACTCACTATAGGGAGGTGCCCATCCTGGTCGAGCT	LC739776.1
ds*EGFP*-T7R	TAATACGACTCACTATAGGGAGTGCACGCTGCCGTCCTCGAT	
q*CAT*-F	TCAGCGTTTGGTGGAGAA	AY518322.1
q*CAT*-R	GCCTGGCTCATCTTTATC	
q*Nrf2*-F	GATGAGAAGCGAGCCAGAGCG	XM_027367068.1
q*Nrf2*-R	GCCGTCGGATGTCTCGGATAA	
q*HSP70*-F	GCGTACTGCCTGTGAGCG	Y645906
q*HSP70*-R	CGGGTGATGGAGGTGTAGAAA	
q*GST*-F	AAGATAACGCAGAGCAAGG	AY573381.2
q*GST*-R	TCGTAGGTGACGGTAAAGA	
q*GPX*-F	AGGGACTTCCACCAGATG	XM_027372127.1
q*GPX*-R	CAACAACTCCCCTTCGGTA	
q*SOD*-F	CTGGTTCCGTTGCTTGGC	DQ005531
q*SOD*-R	CGCTCATTCACGTTCTCCC	
q*Trx*-F	TTAACGAGGCTGGAAACA	XM_027377405.1
q*Trx*-R	AACGACATCGCTCATAGA	
q*HIF*-1*α*-F	GGAGGCCTACAAGACACTGC	FJ807918.1
q*HIF*-1*α*-R	TGAGACACACGACGTACTGC	
q*prx2*-F	AATGACCGCGTTGAGGAGTT	XM_027353910.1
q*prx2*-R	AGTGGGATCTTCAGCTTGCC	
q*ATG5*-F	GGAACCTCACTGCCCACTTT	MH797023.1
q*ATG5*-R	TGCCCTCTGTGCTTCAAACC	
q*ATG10*-F	GGATGCTCTTGTTGCGTCAGG	MH797021.1
q*ATG10*-R	CAATCACTCGGGTAAACTTCT	
q*caspase-2*-F	TAAAGTTCCCTCACGACAA	XM_027358707.1
q*caspase-2*-R	GCTCATCACCATCCCTAAT	
q*caspase-3*-F	AACCAAGGCATCCCTGTCA	XM_027378310.1
q*caspase-3*-R	GGGTTTATTCTGAAGTTGTGGG	
q*EF1α*-F	GTATTGGAACAGTGCCCGTG	XM_027373349.1
q*EF1α*-R	ACCAGGGACAGCCTCAGTAAG	
q*HO-1*-F q*HO-1*-R q*NQO1*-F q*NQO1*-R	GCATGGCAGTGACCGAGATTGA GTCGCTGCTTCGTCTCCTCATC ATGAGTACTTCAGCCAAAATG TCATCATTTTGGCTGAAGTA	XM_027376282.1 XM_027358901.1

## Data Availability

The data that support the findings of this study are available from the corresponding author upon reasonable request.
